# Identification of a lathyrane-type diterpenoid EM-E-11-4 as a novel paclitaxel resistance reversing agent with multiple mechanisms of action

**DOI:** 10.18632/aging.102842

**Published:** 2020-02-28

**Authors:** Qian Liu, Pei Cai, Siwei Guo, Jiangong Shi, Hua Sun

**Affiliations:** 1 State Key Laboratory of Bioactive Substance and Function of Natural Medicines, Institute of Materia Medica, Chinese Academy of Medical Sciences and Peking Union Medical College, Beijing 100050, China; 2 Beijing Neurosurgical Institute, Beijing Tiantan Hospital, Capital Medical University, Beijing 100050, China; 3 Hunan Provincial Maternal and Child Health Care Hospital, Changsha 410008, China

**Keywords:** EM-E-11-4, paclitaxel, multidrug resistance, P-gp, βIII-tubulin

## Abstract

P-glycoprotein (P-gp) and βIII-tubulin overexpression-mediated drug resistance leads to clinical therapy failure for paclitaxel. However, the development of paclitaxel-resistance reversal agents has not had much success. In this study, EM-E-11-4, a lathyrane-type diterpenoid extracted from *Euphorbia micractina*, demonstrated good anti-MDR (multidrug resistance) activity in paclitaxel-resistant tumor cells overexpressing either P-gp or βIII-tubulin. EM-E-11-4 was able to recover the effects of paclitaxel in inducing arrest at G_2_/M phase and apoptosis in both A549/Tax (P-gp overexpression) and Hela/βIII (βIII-tubulin overexpression) cells, respectively, at a non-cytotoxic dose. EM-E-11-4 could enable Flutax-1 and Rhodamine 123 be accumulated intracellularly at an accelerating rate in A549/Tax cells by inhibiting the activity of P-gp ATPase, rather than affecting the expression of P-gp. In addition, it also strengthened the effects of paclitaxel in promoting tubulin polymerization and the binding of paclitaxel to microtubules *in vitro.* It inhibited the expression of βIII-tubulin in Hela/βIII cells in a dose-dependent manner while not exerting influence on the other β-tubulin subtypes. As far as we know, this is the first study to report that a small molecule natural product could specifically inhibit the expression of βIII-tubulin. These results suggest EM-E-11-4 may serve as a promising MDR reversal agent, particularly for patients bearing tumors with high expression of P-gp and βIII-tubulin.

## INTRODUCTION

Chemotherapy is an alternative therapeutic method for patients with cancer. Nevertheless, the outcome is not always satisfactory because drug resistance can develop during the course of treatment. As a first-line chemotherapy, paclitaxel has been used to treat patients suffering from breast and ovarian cancer for decades [1–3]. However, the intrinsic and acquired tumor drug resistance severely restrained its clinical application [4–5], which was observed in cell lines correlated to overexpression of P-glycoprotein (P-gp) and βIII-tubulin [6–8].

As a vital transporter of the ATP-binding cassette (ABC) family [9–10], P-gp is encoded by *abcb1/mdr1* gene [11], functioning as an ATP-dependent broad-spectrum drug efflux pump, and the drug concentration in cells is down-regulated by P-gp. It is involved with lots of structurally uncorrelated anti-cancer drugs, for instance, paclitaxel, docetaxel, doxorubicin, and vincristine [12], and that can lead to multidrug resistance (MDR). The relationship between cancer chemotherapy resistance and P-gp expression has been confirmed in numerous clinical studies [13–15]. In recent years, there has been a certain degree of success in the development of P-gp-mediated paclitaxel resistance reversal agents [16–18].

Many clinical studies have provided evidence that βIII-tubulin overexpression results in another type of paclitaxel resistance in tumor cells [19–21]. Generally, mainly in neuronal cells, βIII-tubulin is expressed and it is rarely detectable in other tissues. However, βIII-tubulin has abnormally high expression in certain drug-resistant tumor cells originating from breast, lung, prostate, and stomach tissues [19, 22]. The exact mechanism of this type of resistance is not fully elucidated.

To overcome the drug resistance mentioned above, our lab pursued novel MDR reversal agents from natural products that can resume the sensitivity to chemotherapy drugs for MDR tumor cells. Among these compounds, EM-E-11-4 is a lathyrane-type diterpenoid from *Euphorbia micractina* that could markedly reverse the sensitivity of drug-resistant cells from different tissues to paclitaxel at its concentration without cytotoxicity. Those tissues we investigated include the human lung adenocarcinoma cell line A549 and its P-gp overexpression drug-resistant counterpart A549/Tax, the same as the paclitaxel-resistant cell line Hela/βIII and the human cervical cell line Hela. Hela/βIII is originated from βIII-tubulin gene transfection. Therefore, we also explored the mechanism of action for the effectiveness of EM-E-11-4 in MDR reversal.

## RESULTS

### Reversal effect of EM-E-11-4 in drug-resistance cells

**Figure 1:**
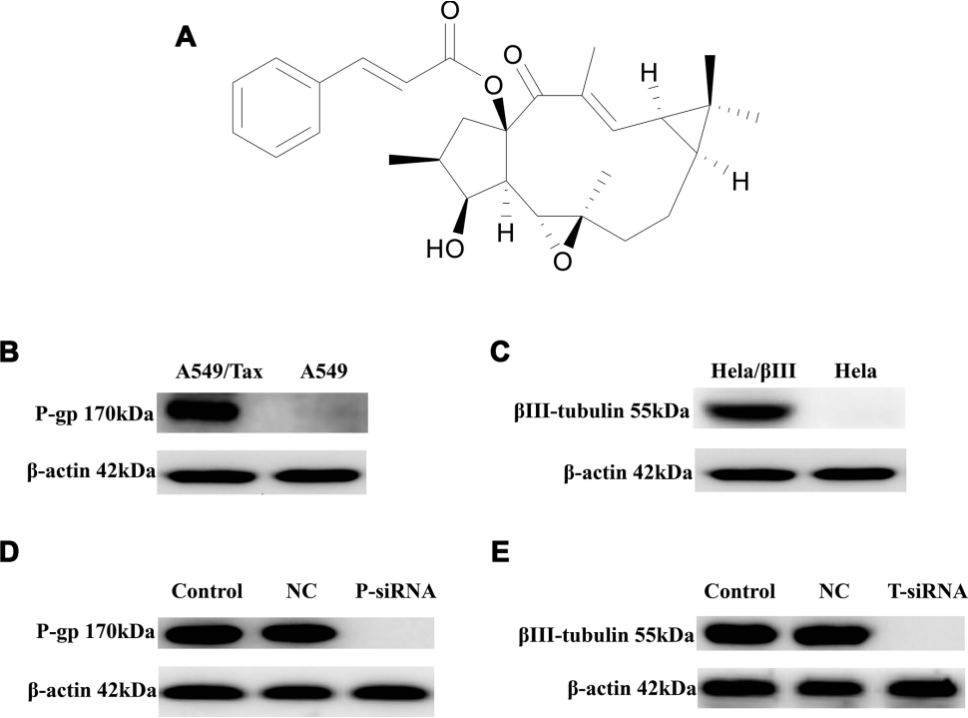
**The expression of P-gp and βIII-tubulin in cells.** (**A**) Chemical structure of EM-E-11-4. (**B**) P-gp levels in A549 and A549/Tax cells. (**C**) βIII-tubulin levels in Hela and Hela/βIII cells. (**D**) The effect of P-gp siRNA on P-gp expression in A549/Tax cells. (**E**) The effect of TUBB3 siRNA on βIII-tubulin expression in Hela/βIII cells. Cells were treated with vehicle (negative control, NC) or siRNAs (P-gp siRNA, TUBB3-siRNA), and protein levels were determined by Western blot analysis.

The cytotoxicity of EM-E-11-4 was examined by an MTT assay in A549/Tax (overexpression of P-gp, Figure 1B) and Hela/βIII (overexpression of βIII-tubulin, Figure 1C) cell lines. As shown in Table 1, EM-E-11-4 at 20-30 μΜ exerted considerable cytotoxicity. In the MDR cell lines, A549/Tax and Hela/βIII demonstrated the same sensitivity to EM-E-11-4 as their parental cells. More than 90% of cells survived at a concentration of 10 μΜ EM-E-11-4 in all assays. According to the results from the cytotoxicity assay mentioned above, EM-E-11-4 at 2.5, 5, and 10 μM was chosen to evaluate the reversal activity. As shown in Table 2, EM-E-11-4 markedly decreased IC_50_ values for paclitaxel in A549/Tax, Hela/βIII, and their parental cells. EM-E-11-4 strengthened the effect of paclitaxel better than verapamil in A549/Tax cells, and it had similar effects in Hela/βIII cells. These results indicate that EM-E-11-4 could reverse paclitaxel-resistance mediated through P-gp or βIII-tubulin.

**Table 1:** Cytotoxic activities of EM-E-11-4 against various human tumor cell lines.

**Compound**	**IC_50_ (μM, Mean ± SD)**		**IC_50_ (μM, Mean ± SD)**	
	**A549**	**A549/Tax**	**Hela**	**Hela/βIII**
EM-E-11-4	31.5 ± 2.3	40.2 ± 2.0	21.1 ± 4.6	25.3 ± 5.8

Data are presented as mean ± SD from three independent experiments.

**Table 2:** Cytotoxic activity of paclitaxel combined with EM-E-11-4 against various human tumor cell lines.

**Cell lines**	**IC_50_ (nM, Mean ± SD)/ Reverse Index**				
	**Paclitaxel**	**+EM-E-11-4 (2.5μM)**	**+EM-E-11-4 (5μM)**	**+EM-E-11-4 (10μM)**	**+Vrp (10μM)**
A549	4.7±1.0	3.3±0.6 (1.4)	0.59±0.08 (8.0)	0.41±0.12 (11.5)	4.3±0.9 (/)
A549/Tax	1559±86	157±13.4 (9.9)	56.7±7.7 (27.5)	22.9 ± 4.7 (68.1)	64.7±7.6 (24.1)
Hela	4.3±0.4	2.8 ± 0.42 (1.5)	1.4±0.3 (3.1)	0.63±0.11 (6.8)	/
Hela/β-III	52.9±4.9	4.6±1.0 (11.5)	3.6±0.8 (14.7)	1.8±0.4 (29.4)	/

Reverse Index = IC_50_ (paclitaxel)/IC_50_ (paclitaxel+EM-E-11-4)

Vrp, verapamil. Data are presented as mean ± SD from three independent experiments.

Through cell transfection with siRNAs targeting P-gp or βIII-tubulin, the expression level of P-gp or βIII-tubulin was suppressed (Figure 1D and 1E). With or without EM-E-11-4 treatment, the activity of paclitaxel in the siRNA transfected cells was respectively assessed. As Table 3 shows, suppressed expression of P-gp or βIII-tubulin through siRNA increases the sensitivity of A549/Tax and Hela/βIII cells to paclitaxel. However, EM-E-11-4 did not dramatically influence the IC_50_ of paclitaxel in those cells. All the results confirm that EM-E-11-4 could reverse drug resistance by suppressing the functions of P-gp or βIII-tubulin.

**Table 3:** Cytotoxic activity of paclitaxel combined with EM-E-11-4 against A549/Tax (P-gp siRNA) and Hela/βIII (TUBB3 siRNA) cell lines.

**Compound**	**IC_50_ (nM, Mean ± SD)**		**IC_50_ (nM, Mean ± SD)**	
	**A549/Tax**		**Hela/βIII**	
	**NC**	**P-siRNA**	**NC**	**T-siRNA**
Paclitaxel	1498±35	24.4±3.6	48.7±4.3	2.4±0.3
Paclitaxel + EM-E-11-4 10μM	25.4±3.2	22.5±2.7	2.1±0.4	2.3±0.4

Data are presented as mean ± SD from three independent experiments.

### EM-E-11-4 strengthened the effect of paclitaxel-induced G_2_/M phase arrest and apoptosis in A549/Tax and Hela/βIII cells

**Figure 2:**
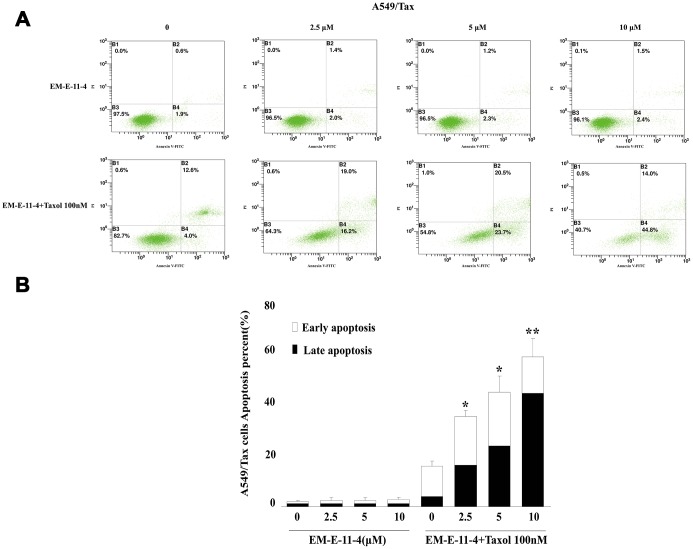
**Apoptosis as detected by Annexin V-FITC/PI binding assay (48 h) in A549/Tax cells.** (**A**) Apoptosis induction by EM-E-11-4 (2.5, 5, and 10 μM) combined with or without 100 nM paclitaxel in A549/Tax cells. (**B**) The percent apoptosis in A549/Tax cells. Columns represent the means±SD values for apoptotic cells obtained from three individual experiments. * *p*<0.05 and ** *p*<0.01 vs. control (10 μM EM-E-11-4).

**Figure 3:**
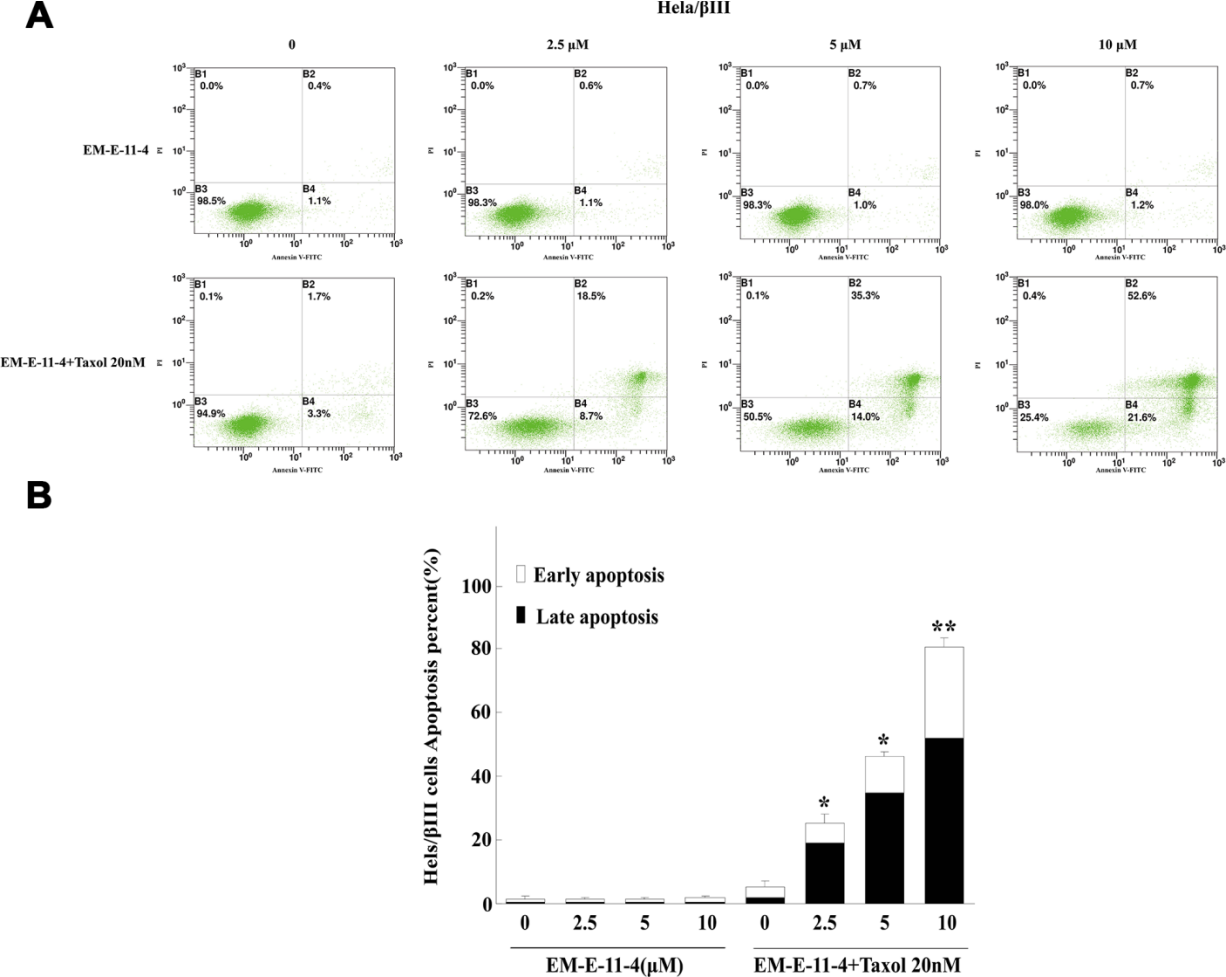
**Apoptosis detected by Annexin V-FITC/PI binding assay (48 h) in Hela/βIII cells.** (**A**) Apoptosis induction by EM-E-11-4 (2.5, 5, and 10 μM) combined with or without 20 nM paclitaxel in Hela/βIII cells. (**B**) The percent apoptosis of Hela/βIII cells. Columns represent the means±SD values for apoptotic cells obtained from three individual experiments. * *p*<0.05 and ** *p*<0.01 vs. control (10μM EM-E-11-4).

It is well known that paclitaxel can trigger cell arrest in G_2_/M phase and apoptosis by disturbing microtubule function, whereas the overexpression of βIII-tubulin and P-gp suppressed these effects. A549/Tax and Hela/βIII cells were treated with paclitaxel (A549/Tax: paclitaxel 100 nM; Hela/βIII: paclitaxel 20 nM) alone or in combination with EM-E-11-4 for 24 h (Supplementary Figure 1 and Supplementary Figure 2). EM-E-11-4 did not affect the cell cycle of A549/Tax and Hela/βIII cells alone. However, paclitaxel combined with EM-E-11-4 (2.5, 5, and 10 μM) increased the percentage of G_2_/M phase from 31.7% (100 nM paclitaxel alone) to 42.7%, 45.7%, and 60.5% in A549/Tax cells (Supplementary Figure 1). It increased from 40.1% (20 nM paclitaxel alone) to 52.7%, 53.7%, and 58.1% in Hela/βIII cells (Supplementary Figure 2). At the same time, EM-E-11-4 increased the percentage of paclitaxel-induced apoptosis for 48 h. Paclitaxel combined with EM-E-11-4 (2.5, 5, and 10 μM) increased the percentage of apoptotic cells from 21.9% (100 nM paclitaxel alone) to 34.6%, 40.2%, and 44.2% in A549/Tax cells (Figure 2). It increased from 5.23% (20 nM paclitaxel alone) to 27.76%, 49.08%, and 76.92% in Hela/βIII cells (Figure 3). These results indicate that EM-E-11-4 could enhance the effect of paclitaxel-induced G_2_/M phase arrest and apoptosis in cells overexpressing P-gp and βIII-tubulin in a dose-dependent manner.

### EM-E-11-4 accelerated the accumulation of Flutax-1 and Rhodamine 123 in A549/Tax cells

**Figure 4:**
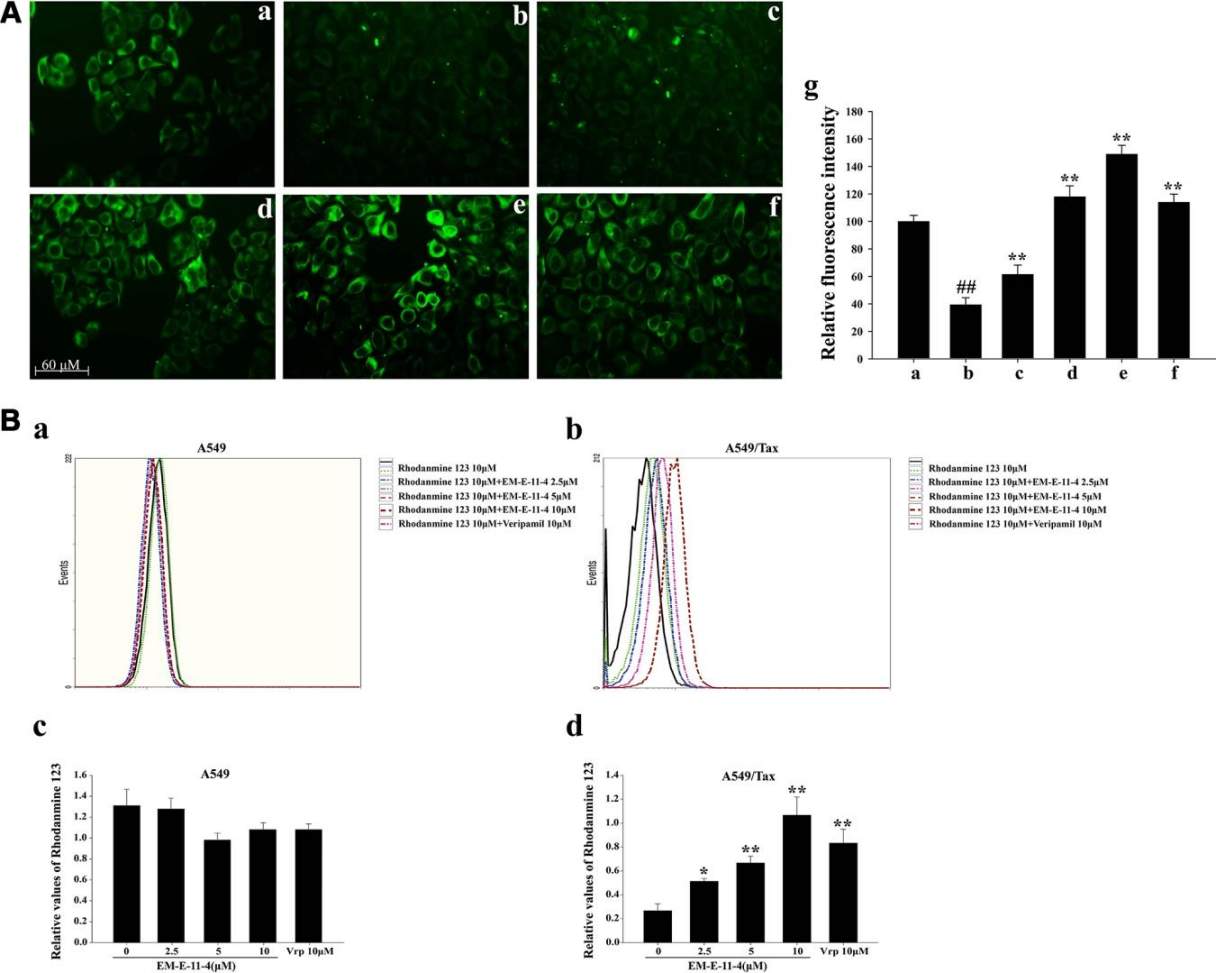
**Effects of EM-E-11-4 on the accumulation of Flutax-1 and Rhodamine 123 in A549 and A549/Tax cells.** (**A**) Effects of EM-E-11-4 on the accumulation of Flutax-1 in A549 and A546/Tax (×100) cells. (**A**-**a**) 5 μM Flutax-1 in A549 cells; (**A**-**b**) 5 μM Flutax-1 in A549/Tax cells; (**A**-**c**) 5 μM Flutax-1 and 2.5 μM EM-E-11-4 in A549/Tax cells; (**A**-**d**) 5 μM Flutax-1 and 5 μM EM-E-11-4 in A549/Tax cells; (**A**-**e**) 5 μM Flutax-1 and 10 μM EM-E-11-4 in A549/Tax cells; (**A**-**f**) 5 μM Flutax-1 and 10 μM verapamil in A549/Tax cells; (**A**-**g**) quantitative data for the fluorescence intensity. ## *p*<0.01 vs. A549 cells control, ** *p*<0.01 vs. A546/Tax cells control. (**B**) Effects of EM-E-11-4 ond the accumulation of Rhodamine 123 in A549 and A549/Tax cells. (**B**-**a**) The levels of Rhodamine 123 assayed by flow cytometry in A549 cells; (**B**-**b**) The levels of Rhodamine assayed by flow cytometry in A549/Tax cells; (**B**-**c**) The relative values of Rhodamine 123 in A549 cells; (**B**-**d**) The relative values of Rhodamine 123 in A549/Tax cells. Columns represent the means±SD values for Rhodamine 123 obtained from three individual experiments. * *p*<0.05 and ** *p*<0.01 vs. control (10μM EM-E-11-4).

P-gp is characterized by a decrease in accumulation and enhancement of the efflux of anticancer drugs, whose effects can be reversed through P-gp inhibitors, for example, verapamil. To investigate the mechanism in which EM-E-11-4 reverses P-gp-mediated paclitaxel resistance in tumors, a P-gp overexpressing cell line, A549/Tax, was used as the drug-resistant model. Flutax-1 is a taxane coupled with a fluorescent group that can bind to the taxol-binding site of β-tubulin and be used as a P-gp substrate. After incubation with 5μM Flutax-1 alone or in combination with EM-E-11-4 for 3 h, A549 and A549/Tax cells were observed by fluorescence microscope (Figure 4A). A549 cells exhibited a normal network of microtubules (MTs) with clear green fluorescence, which indicates that Flutax-1 entered the cell and bound to the taxol-binding site of MTs (Figure 4A-a). A549/Tax cells exhibited weak fluorescence, which indicates that P-gp mediates the efflux of Flutax-1 (Figure 4A-b). EM-E-11-4 dose-dependently increased Flutax-1 accumulation in A549/Tax cells (Figure 4A-c, d and e). After treated A549/Tax cells with Flutax-1 and 10 μM EM-E-11-4, they exhibited network of MTs that seemed clearer than the parallel assay using 10 μM verapamil (Figure 4A-f). After treatment with Rhodamine 123 (Rh123) and EM-E-11-4 for 0.5 h in A549 and A549/Tax cells, the level of intracellular Rh123 was monitored using flow cytometry (Figure 4B). In A549 cells, EM-E-11-4 made no difference on the concentration of intracellular Rh123 (Figure 4B-a and c). However, in A549/Tax cells, EM-E-11-4 dose-dependently increased the level of intracellular Rh123 (Figure 4B-b and d). These results suggest EM-E-11-4 could reverse P-gp-mediated drug resistance through suppressing the efflux function of P-gp, and it might accelerate the accumulation of paclitaxel in A549/Tax cells.

### EM-E-11-4 inhibits the ATPase activity of P-gp without affecting the expression of P-gp

**Figure 5:**
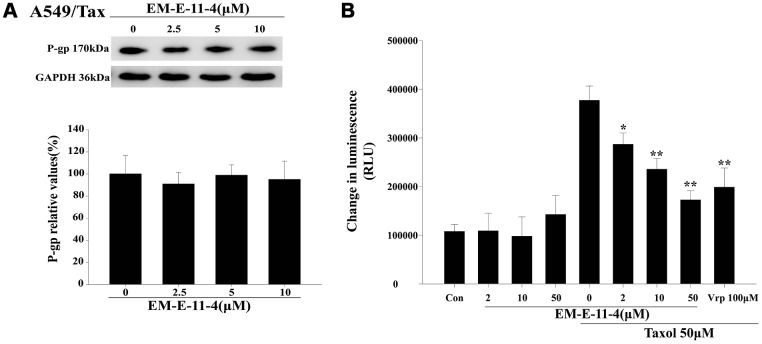
**EM-E-11-4 inhibited the P-gp ATPase activity.** (**A**) A549/Tax cells were treated with EM-E-11-4 (2.5, 5, and 10 μM) for 48 h, and P-gp was determined by Western blot analysis. (**B**) A luminescent assay for P-gp ATPase activity was performed according to the Pgp-Glo^TM^ Assay System instructions. ATP consumption in the presence EM-E-11-4 (2, 10, and 50 μM) combined with or without paclitaxel. Con: control; Vrp: verapamil. Columns represent the means±SD values for the luminescent change obtained from three individual experiments * *p*<0.05 and ** *p*<0.01 vs. control (10μM EM-E-11-4).

After treated A549/Tax cells with EM-E-11-4 for 48 h, the expression level of P-gp had no marked change (Figure 5A). However, EM-E-11-4 did affect the ATPase activity of P-gp in the Pgp-Glo^TM^ Assay System. As a substrate of P-gp, paclitaxel binds to P-gp and is transported through the protein. Thus, with the increasing consumption of ATP, which was assessed as a change in luminescence, the ATPase activity was demonstrated to be increase. Na_3_VO_4_ inhibits the P-gp ATPase activity, and with the existence of Na3VO4, the data were normalized refer to the ATP consumption. As Figure 5B shows, small change in luminescence was detected with the untreated vehicle control and EM-E-11-4 (2, 10, and 50 μM), which indicates low ATP consumption. This reflects the weak intrinsic basal ATPase activity of P-gp. Adding paclitaxel (50 μM) led to increasingly higher ATP consumption, which was reflected in the stimulation of P-gp ATPase activity. With the existence of paclitaxel combined with EM-E-11-4 (2, 10, and 50 μM), ATP consumption was actually lower than in the presence of paclitaxel alone, which indicates a lower level of ATPase consumption. With the existence of paclitaxel combined with 50 μM EM-E-11-4, ATP consumption was lower than when it was combined with 100 μM verapamil. These results suggest that EM-E-11-4 hinders P-gp ATPase activity that results in a decrease in the efflux of intracellular paclitaxel by P-gp. The results partly illustrate its circumvention function of P-gp-mediated drug resistance.

### EM-E-11-4 strengthened the effect of paclitaxel-induced tubulin polymerization *in vitro*

**Figure 6:**
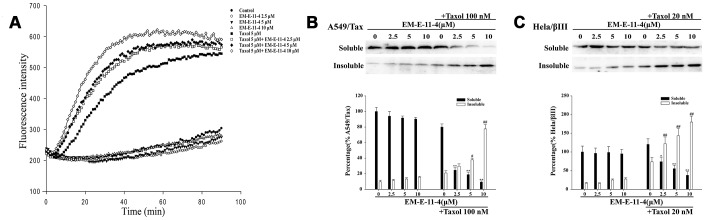
**EM-E-11-4 enhanced the effect of paclitaxel-induced tubulin polymerization *in vitro*.** (**A**) Tubulin polymerization assay. Purified porcine tubulin in reaction buffer was incubated at 37°C with GTP in the absence or presence of the indicated agents. Tubulin polymerization was measured using a fluorescence microplate reader (ex = 370 nm, em = 445 nm) every 1 min for 60min. (**B**) A549/Tax cells were treated with EM-E-11-4 (2.5, 5, and 10 μM) and/or 100 nM paclitaxel for 48 h. Then, the soluble tubulin and insoluble tubulin were isolated, and the levels of α-tubulin were determined the by Western blot analysis. (**C**) Hela/βIII cells were treated with EM-E-11-4 (2.5, 5, and 10 μM) and/or 100 nM paclitaxel for 48 h. Then, the soluble tubulin and insoluble tubulin were isolated, and the levels of α-tubulin were determined by Western blot analysis. Columns represent the means±SD values for protein levels obtained from three individual experiments. * *p*<0.05 and ** *p*<0.01 vs. Soluble control (A: paclitaxel 100 nM; B: paclitaxel 20 nM), # *p*<0.05 and ## *p*<0.01 vs. Insoluble control (A: paclitaxel 100 nM; B: paclitaxel 20 nM).

As shown in Figure 6, paclitaxel could promote tubulin polymerization *in vitro* since the tubulin polymerization levels are reflected by the fluorescence intensity. EM-E-11-4 dose-dependently strengthened the effect of paclitaxel-induced tubulin polymerization, whereas EM-E-11-4 alone did not induce tubulin polymerization (Figure 6A). Western blot analysis indicated that EM-E-11-4 has no influence on the soluble and insoluble states of MTs in A549/Tax and Hela/βIII cells. However, EM-E-11-4 combined with paclitaxel markedly increased the proportion of MTs in the insoluble states more than paclitaxel alone (Figure 6B and 6C). Our study also shows that EM-E-11-4 promotes binding constant of paclitaxel for MTs (Supplementary Table 1). These results indicate EM-E-11-4 was capable of enhancing the effect of paclitaxel-induced tubulin polymerization and the binding of paclitaxel to MTs. It is one of the reasons why it strengthened the effect of paclitaxel-induced G_2_/M phase arrest and apoptosis, causing reverse drug resistance in cancer cells.

### EM-E-11-4 inhibited the expression of βIII-tubulin in Hela/βIII cells

**Figure 7:**
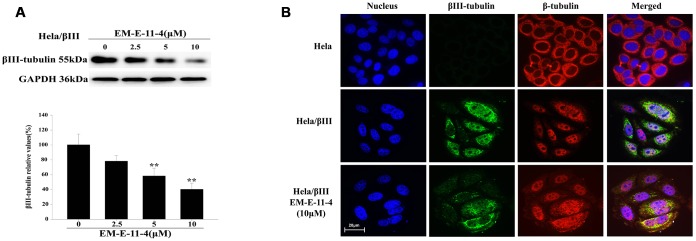
**EM-E-11-4 inhibited the expression of βIII-tubulin.** (**A**) Hela/βIII cells were treated with EM-E-11-4 (2.5, 5, and 10 μM) for 48 h, and the expression of βIII-tubulin was determined by Western blot analysis. Columns represent the means±SD values for protein levels obtained from three individual experiments. * *p*<0.05 and ** *p*<0.01 vs. control (EM-E-11-4 0μM). (**B**) Immunofluorescence analysis (×600). Blue: nucleus; green: βIII-tubulin; red: β-tubulin. There is no clear green fluorescence in Hela cells, whereas Hela/βIII cells have a clear green fluorescence. After Hela/βIII cells were treated with EM-E-11-4 48 h, the green fluorescence was weakened.

We found EM-E-11-4 decreases the level of βIII-tubulin expression in Hela/βIII cells (Figure 7). After treating Hela/βIII cells with EM-E-11-4 for 48 h, Western blot (Figure 7A) and immunofluorescence analysis (Figure 7B) indicated that the expression of βIII-tubulin was decreased by EM-E-11-4 in a dose-depend manner. This suggests that inhibition of βIII-tubulin expression was the most important function of EM-E-11-4 to overcome βIII-tubulin-mediated paclitaxel resistance in tumors.

### Molecular docking analysis of EM-E-11-4 to determine the activity capacity of TUBB3 and P-gp

**Figure 8:**
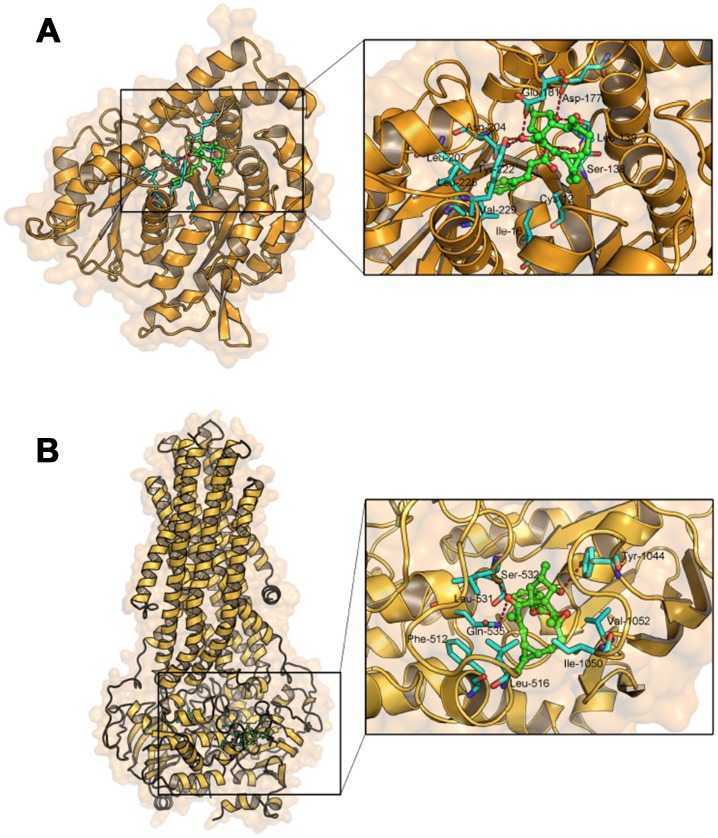
Molecular docking analysis of EM-E-11-4 to the activity cavity of TUBB3 (**A**) and P-gp (**B**).

We investigated the interactions between EM-E-11-4 and TUBB3 or P-gp by molecular docking analysis. The docking results revealed that EM-E-11-4 forms six hydrogen bonds with the residues of Ser-138, Leu-139, Asp-177, Glu-181, Asn-204, and Tyr-222 in TUBB3 (Figure 8A). In addition, the hydrophobic group of EM-E-11-4 inserts into a hydrophobic gap and interacts with Cys-12, Ile-16, Leu-207, Leu-225, and Val-229. Similarly, EM-E-11-4 and P-gp form three hydrogen bonds with the residues of Ser-532, Gln-535, and Tyr-1044, and the hydrophobic groups interact with Phe-512, Leu-516, Leu-531, Ile-1050, and Val-1052 (Figure 8B). Furthermore, the mode of binding in both TUBB3 and P-gp show significant similarity. The macrolide was exposed to the solvent region and formed stable hydrogen bonds and hydrophobic interactions. The phenyl group inserts into the deep hydrophobic gap to form stable hydrophobic interactions. To sum up, critical hydrogen bonds and hydrophobic interactions between EM-E-11-4 and TUBB3 or P-gp were predicted by molecular docking, indicating the EM-E-11-4 may server as a potent agent by simultaneously targeting TUBB3 and P-gp.

## DISCUSSION

As a first-line drug for various cancer treatment, paclitaxel is frequently used as chemotherapeutic agents. However, paclitaxel has been greatly limited in helping cure cancer byβIII-tubulin and P-gp-mediated drug resistance [6–8]. Originated from *Euphorbia micractina*, EM-E-11-4 is a lathyrane-type diterpenoid. Preliminary activity screening results have shown that it could enhance the effects of chemotherapeutic agents, especially paclitaxel, in drug-resistant cancer cells (data not shown). Our study found EM-E-11-4 could markedly enhance paclitaxel cytotoxicity in A549/Tax (P-gp overexpression) and Hela/βIII (βIII-tubulin overexpression) cells.

As an ATP-binding cassette transporter, P-gp has been extensively studied for more than 20 years. The overexpression of P-gp is a main mechanism referred to paclitaxel resistance in tumors. It can decrease the accumulation of paclitaxel in cells, which leads to its chemotherapeutic failure. Therefore, inhibition of its expression or its transporting function could reverse P-gp-mediated drug resistance. Many P-gp inhibitors have been developed and dedicated to clinical studies [23], such as verapamil (the 1^st^-generation nonspecific P-gp inhibitor) [24], valspodar, biricodar (the 2^nd^-generation P-gp inhibitor with more potent and more selectivity) [25, 26], and tariquidar (the 3^rd^-generation P-gp inhibitor with the most potent selectivity) [27].

P-gp inhibition mainly have 3 mechanisms as follows: (a) directly affecting the drug-binding sites of P-gp (competitive inhibitor); (b) affecting ATP binding and hydrolysis, which affects the function of P-gp (noncompetitive inhibitor) [28–29]; (c) affecting the allosteric pocket residues, which inhibits P-gp activity and translocation [30]. EM-E-11-4 was able to up-regulate the accumulation of Flutax-1 and Rhodamine 123 in A549/Tax cells by suppressing the ATPase activity of P-gp rather than changing P-gp expression levels. Based on the mechanisms mentioned above, EM-E-11-4 could be indicated as a noncompetitive inhibitor of P-gp, and thus, it could reverse P-gp-mediated drug resistance in tumors.

Microtubule stabilizing agents (MSA) bind to the tubulin polymer that stabilize MTs and inhibit MT depolymerization, resulting in mitotic arrest (G_2_/M phase arrest) and cell apoptosis [31, 32]. EM-E-11-4 strengthened the effect of paclitaxel-induced tubulin polymerization and promoted tubulin from soluble states to insoluble states. It also increased the binding of paclitaxel to MTs. Therefore, it strengthened the effect of paclitaxel-induced cell arrest in G_2_/M phase and apoptosis in drug-resistant cancer cells. This is a mechanism in which EM-E-11-4 reverses paclitaxel resistance in cancer cells. J. Zhou et al found that the compound EM012 strengthened the anti-proliferative activity of paclitaxel, which originated from its activity of synergistic/additive inhibition of microtubule dynamics [33]. We speculate that EM-E-11-4 may bind near the paclitaxel-binding domain, which leads to a conformation change in the protein. This enhances the ability of paclitaxel to promote tubulin polymerization and bind to MTs.

The relevance of βIII-tubulin overexpression with paclitaxel resistance in tumors has been confirmed within the last 10 years [32]. However, the mechanism of βIII-tubulin-mediated tumor drug resistance has not been completely elucidated yet. Previous studies have found that βIII-tubulin can enhance the dynamic nature of MTs. Therefore it counteracts the stabilization effect of MT-interacting agents, for example, paclitaxel [34]. Also, the amino acid sequence is different from other β-tubulin isotypes at the paclitaxel-binding domain, which may influence paclitaxel binding to β-tubulin in MTs [35, 36]. βIII-tubulin was also reported as a factor in cell survival. It was found that βIII-tubulin could induce resistance to other drugs in addition to paclitaxel, or it could induce tumor survival in an abnormal environment [37]. At present, there is limited research on βIII-tubulin inhibition by the use of siRNAs or miRNAs to silence βIII-tubulin at the cellular level, and they are without a breakthrough [37–39]. Until now, there has not been a small molecular compound found that is able to inhibit the expression of βIII-tubulin. Our results confirm that EM-E-11-4 inhibits βIII-tubulin expression while doesn’t affect the other β-tubulin subtypes (data not shown). EM-E-11-4 was indicated as a specific inhibitor of βIII-tubulin. This might be the most important reason that EM-E-11-4 can significantly reverse βIII-tubulin-mediated paclitaxel resistance in Hela/βIII cells.

In addition, we investigated the interactions between EM-E-11-4 and TUBB3 or P-gp by molecular docking analysis. Interestingly, the mode of binding in both TUBB3 and P-gp demonstrated significant similarity. Critical hydrogen bonds and hydrophobic interactions between EM-E-11-4 and TUBB3 or P-gp were predicted by molecular docking. A highly similar mode of binding may indicate a similar mechanism of EM-E-11-4 interfering with the function of P-gp and βIII-tubulin in paclitaxel resistant tumor cells.

To summarize, our study has demonstrated that EM-E-11-4 could reverse P-gp and βIII-tubulin-mediated paclitaxel resistance in tumor cells. It suppressed the ATPase activity of P-gp, which decreases the efflux of paclitaxel by P-gp for the lack of energy. At the same time, it inhibited the expression of βIII-tubulin, resulting in a strengthened effect of paclitaxel in Hela/βIII cells. These results suggest that EM-E-11-4 may serve as an efficient MDR reversal agent, particularly for patients with high expression of P-gp or βIII-tubulin in tumors.

## MATERIALS AND METHODS

### Materials

Paclitaxel was obtained from Beijing Union Pharmaceutical Factory, with purity over 99%. EM-E-11-4 was isolated from *Euphorbia micractina*, with purity over 99%. Flutax-1 and Flutax-2 were synthesized in our lab, with purity over 99%. The chemical structure of EM-E-11-4 showed in (Figure 1A). Compounds dissolved in dimethyl sulphoxide before use. Rhodamine 123 (Rh123), Verapamil (Vrp), MTT, PI, and RNase A were purchased from Sigma-Aldrich (St. Louis, MO, USA). Fetal bovine serum (FBS), DMEM nutrient mixture and RPMI 1640 were obtained from Gibco BRL (Grand Island, NY, USA). TUBB3 siRNA (50-UCUCUUCAGGCCUGACAAUTT-30), P-gp siRNA (50-GCGAAGCAGUGGUUCAGGUTT-30), the negative control (50-UUCUCCGAACGUGUCACGUTT-30) [40] and the Lipofectamine RNAiMAX Reagent were obtained from Invitrogen Trading Co., Ltd. (Shanghai). Apart from the antibodies for β-tubulin, α-tubulin, βIII-tubulin and P-gp which were obtained from Abcam (UK), all other antibodies were obtained from Cell Signaling Technology (USA). From Cytoskeleton (USA), we obtained Tubulin Polymerization Assay Kit (Porcine tubulin fluorescence based, Cat.#BK011P) while Pgp-Glo™ Assay Systems was purchased from Promega (USA). The Annexin V-FITC/PI Apoptosis Kit was purchased from Beyotime Institute of Biotechnology (China). The 4’, 6-diamidino-2-phenylindole (DAPI) dihydrochloride nuclear stain, Texas Red® goat anti-rabbit IgG antibody and Alexa Fluor goat anti-mouse IgG antibody were provided from Invitrogen (Carlsbad, CA).

### Cell lines and culture

The human lung cancer cell line A549 and its paclitaxel-resistant counterpart A549/Tax were supplied by Laboratory of Pharmacology, Institute of Materia Medica, Chinese Academy of Medical Sciences and Peking Union Medical Collage. Hela and Hela/βIII cells were provided from Dr. Richard Ludeña at the University of Texas. A549 and A549/Tax cells were grown in medium RPMI 1640 including 10% FBS, supplemented with 100 μg/mL streptomycin and 100 units/mL penicillin. The other cell lines were cultured in DMEM including the same proportion and amount of FBS, streptomycin and penicillin. Drugs (A549/TAX: 10 nM TAX, Hela/βIII: 0.5 mg/mL G418 sulfate) were added to the counterpart drug-resistant cell lines, and the medium containing drugs was replaced with the normal medium a week before the experiments. All cells were cultured with 5% CO2 in air at 37°C.

### MTT assay

Using MTT assay, cell survival rate after EM-E-11-4 and drug treatment was evaluated. Briefly, 5000 cells/well were seeded in 96-well plates, and then 24h later, the cells were treated with the test agent at different concentrations for 3 d. Then, 20 μL of 5 mg/mL MTT was added to the wells for 4 h at 37 °C. Next, removed the medium and added 150μL of DMSO to every well. A microplate reader (490 nm) was given to test the absorbance. The IC_50_ values for drugs were analyzed by SPSS 17.0.

### siRNA transfection

Cells (5×10^4^) were seeded in 6-well cell culture plates and cultured for 24 h. Then cells were transfected with siRNAs (TUBB3 siRNA/P-gp siRNA: 100 nM) [40]. After 48 h, used western blotting examined the expression of P-gp and βIII-tubulin. siRNA-transfected cells (3×10^3^) were exposed to different concentrations of compounds. The IC_50_ of Taxol with or without EM-E-11-4 in siRNA-transfected cells were analyzed by MTT assay.

### *In vitro* tubulin polymerization assay

Using a Tubulin Polymerization Assay Kit to analyze the capability of EM-E-11-4 and paclitaxel to promote tubulin polymerization. Briefly, tubulin proteins (2 μg/μL) were suspended in G-PEM buffer (pH 6.9, 2 mM MgCl_2_, 80 mM PIPES, 1.0 mM GTP, 0.5 mM EGTA and 15% glycerol), and the test agents were added at 4 °C. The sample mixture was transferred to 96-well plates (37°C), and the variations of fluorescence intensity (ex=370 nm, em=445 nm) were measured for 1.5 h.

### Immunofluorescence assay

Hela/βIII cells (3×10^3^) were grown on fibronectin-coated cell culture dishes, and then incubated with or without 10 μΜ EM-E-11-4 for 48 h. Then cells were diluted with PBS, fixed in 4% paraformaldehyde (20 min) and blocked with 5% goat serum in 0.1% Triton X-100 in PBS for 10 min at room temperature. The cells were incubated with anti-βIII-tubulin and anti-β-tubulin antibody (1:100) at 4°C overnight. They were then incubated with the secondary antibody (Texas Red® goat anti-rabbit IgG antibody, Alexa Fluor goat anti-mouse IgG antibody) for 1 h, and then they were incubated with DAPI for 5 min in the dark. Images were examined using a PerkinElmer UltraVIEWVoX system (PerkinElmer Life Sciences Inc., MA, USA).

### P-gp ATPase activity assay

ATPase activity of P-gp was conducted by the Pgp-Glo^TM^ assay system conforming to the instructions. Following incubation with EM-E-11-4 and paclitaxel, the luminometer was used to measure the luminescence of the P-gp ATPase reaction system. The luminescence represents ATP contents, which are negatively related to the P-gp ATPase activity. This is indicative of the capability of P-gp-mediated transport.

### The accumulation of Flutax-1

Flutax-1 is a taxane tagged with a fluorescent group, and it is a fluorescent probe that can selectively combined in taxol-binding site of β-tubulin. Cells (5×10^5^) were seeded in 6-well plates. Incubation with Flutax-1 and compounds for 3 h, cells were then diluted once with PBS, then observed with a fluorescence microscope.

### The accumulation of Rhodamine 123 (Rh123)

Using flow cytometry (Sigma, USA) determined the fluorescence intensity of Rh123 in cells. Briefly, cells (5×10^5^) were seeded in a 6-well plate and incubated with compounds for 30 min. The cells were harvested, then detected the fluorescence intensity of Rh123 in cells (ex=475nm, em=525nm) by utilizing flow cytometry.

### Analysis of cell cycle

Flow cytometry was used to detect cell cycle distribution. Cells (5×10^5^) were seeded in 6-well plates and incubated with compounds for 1 d before the cells were extracted and immersed in 70% ethanol. And cells were incubated with RNase A solution (50 μg/mL) and stained with propidium iodide (50 μg/mL) for 30 min in the dark. The samples were examined with flow cytometry.

### Apoptosis assay

The Annexin V-FITC Apoptosis Detection Kit was used for quantitative determination of apoptotic cells. After incubation with the test agents for 48 h, cells were collected, washed with PBS, and then suspended in 100 μL of binding buffer. A total of 5 μL of Annexin V-FITC was added to the suspension and incubated for 10 min at 25°C. Then, 10 μL of PI was added to the cells and incubated for another 15 min in the dark. Then, 400 μL binding buffer was added prior to analysis by flow cytometry. Early apoptosis is defined the cells in the second phase; Late apoptosis is defined the cells in the third phase.

### Cell tubulin polymerization assay [41]

Soluble (cytosolic) and insoluble (cytoskeletal) tubulin from cell lysates were separated as previously described. Briefly, A549/Tax and Hela/βIII cells were incubated with EM-E-11-4 (2.5, 5, 10 μM) and paclitaxel (A549/Tax: 100 nM; Hela/βIII: 20 nM) for 48 h. Subsequently, cells were lysed at 37°C with 150 μL of hypotonic buffer [41]. The fractions were separated by centrifugation at a speed of 12,000 rpm/20 min at 25°C. The supernatant fraction inclusive of soluble tubulin was transferred to another tube. The precipitation fraction inclusive of insoluble tubulin resuspended in the same buffer. SDS-PAGE extracted and resolved proteins.

### The determination of paclitaxel binding constants for MTs

The binding constants of paclitaxel to the paclitaxel binding site of microtubules (MTs) were assessed as previously mentioned [42]. Briefly, Flutax-2 is a taxane with a fluorescence group that can be used as a fluorescent probe to reversibly bind to a taxol-binding site. When paclitaxel was added to the MTs and Flutax-2 mixture, it competitively binds to the taxol-binding site, which leads to Flutax-2 dissociating from MTs. Free Flutax-2 changes the fluorescence polarization value of the reacting system, which calculates the concentration of binding-paclitaxel. Then, according to the binding constants of Flutax-2 for MTs, the binding constants of paclitaxel were calculated.

### Western blot analysis

Cells were treated with test agents and then lysed in denaturing lysis buffer (1 mM MgCl_2_,20 mM Tris-HCI PH 6.8, 2 mM EGTA, 2 μg/mL Pepstatin, l% NP40, 2 μg/mL Aprotinin, 2 mM PMSF). The samples were isolated by SDS–PAGE, transferred to PVDF membranes. Then membranes were blocked with 5% BSA in TBST for 60 min at 25°Cprior to incubation with antibodies against P-gp, β-actin, βIII-tubulin, α-tubulin, and GAPDH overnight at 4°C. Incubated the membranes with their corresponding secondary antibody for 60 min at 25°C, and they were visualized through enhanced chemiluminescence system (GE Healthcare, USA).

### Molecular docking analysis

To probe the interactions between EM-E-11-4 and TUBB3 or P-gp, a molecular docking study was performed using AutoDock (version 4.2.6) [43]. Based on a Lamarkian genetic algorithm, AutoDock package is a flexible docking program for searching the best conformation of the ligand in a macromolecule. The crystal structure of human TUBB3 [44] (PDB code: 5IJ0) and P-gp [45] (PDB code: 6C0V) were derived from the Protein Data Bank. AutoDockTools (version 1.5.6) was utilized for generation of the docking input files [44]. A grid box size of 50 × 50 × 50 points with a 0.375 Å spacing between the grid points was applied. Affinity maps of TUBB3 and P-gp were computed through AutoGrid. The Lamarckian genetic algorithm (LGA) was directed at determination of EM-E-11-4 and protein interaction. The docking parameters are described below: trials of 100 dockings, and the number of individuals in the population were set as 150 and 250,000 energy evaluations. All other settings were left as they were by default. To analyze the docking results, AutoDockTools version 1.5.6 and PyMol came into use [43, 46].

### Statistical analysis

All data were expressed as the mean ± standard deviation (SD) of at least three independently performed experiments. The statistical significance of the differences among three groups was analyzed by one-way analysis of variance (ANOVA), which was followed by a least significant difference post-hoc test to obtain individual P values. The Student’s t-test was used to determine differences between the two groups. A value p <0.05 was considered statistically significant.

## Supplementary Material

Supplementary Figures

Supplementary Table 1
