# Supplementary material for: Identification of a lathyrane-type diterpenoid EM-E-11-4 as a novel paclitaxel resistance reversing agent with multiple mechanisms of action
Source: Aging (Albany NY). 2020 Feb 28;12(4):3713–29. doi: 10.18632/aging.102842 (PMC7066893; doi:10.18632/aging.102842)
Supplement: Supplementary Figures [file aging-12-102842-s002..pdf]

SUPPLEMENTARY FIGURES

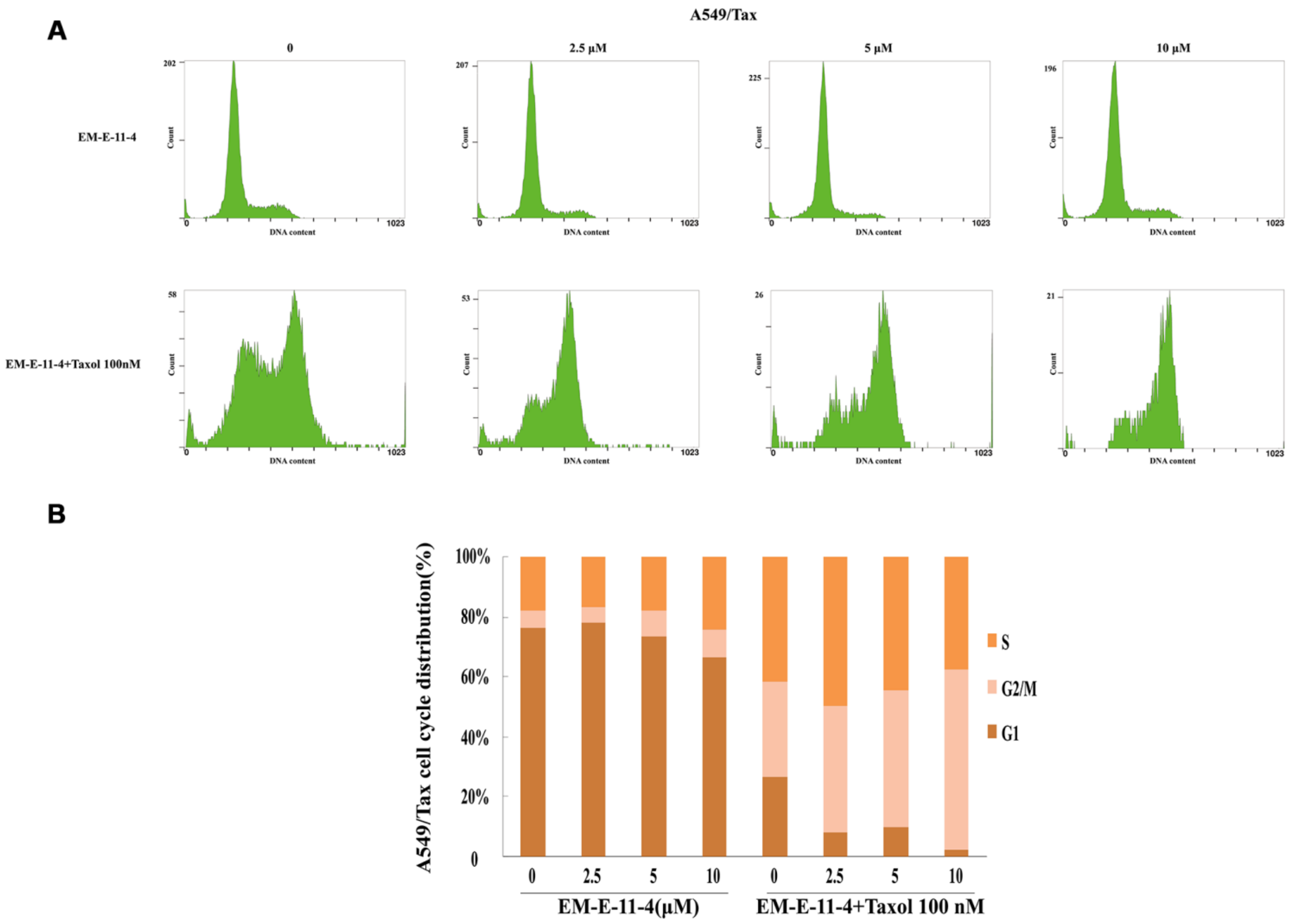

Supplementary Figure 1. G_2/M phase arrest induced by EM-E-11-4 and paclitaxel (24 h) in A549/Tax cells. (**A**) Cell cycle effects of EM-E-11-4 (2.5, 5, and 10 µM) combined with or without 100 nM paclitaxel in A549/Tax cells. (**B**) The cell cycle distribution of A549/Tax cells.

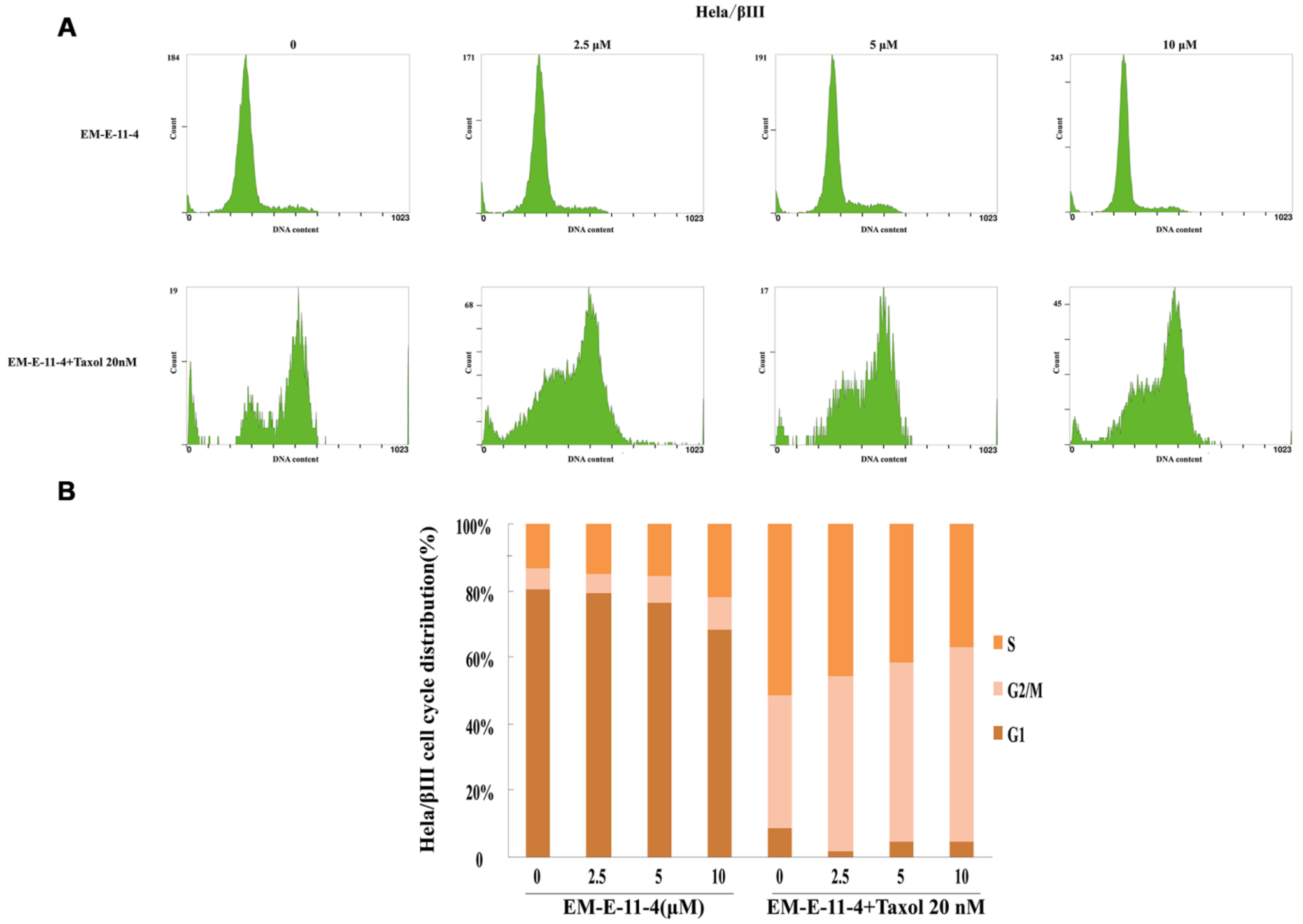

Supplementary Figure 2. G_2/M phase arrest induced by EM-E-11-4 and paclitaxel (24 h) in Hela/βIII cells. (**A**) Cell cycle effects of EM-E-11-4 (2.5, 5, and 10 μM) combined with or without 20 nM paclitaxel in Hela/βIII cells. (**B**) The cell cycle distribution of Hela/βIII cells.
