# Supplementary material for: Identification of a lathyrane-type diterpenoid EM-E-11-4 as a novel paclitaxel resistance reversing agent with multiple mechanisms of action
Source: Aging (Albany NY). 2020 Feb 28;12(4):3713–29. doi: 10.18632/aging.102842 (PMC7066893; doi:10.18632/aging.102842)
Supplement: Supplementary Table 1 [file aging-12-102842-s001..pdf]

SUPPLEMENTARY TABLE

Supplementary Table 1. The binding constants of paclitaxel for MTs (35°C).

Compound	Binding constants of paclitaxel
/	0.52±0.05 M^{-1}
+ EM-E-11-4 10μM	1.59±0.07 M^{-1}

Data are presented as mean ± SD from three independent experiments.
